# (*E*)-Ethyl 2-cyano-2-(thia­zolidin-2-yl­idene)acetate

**DOI:** 10.1107/S1600536808032984

**Published:** 2008-10-18

**Authors:** Jian-Gang Wang, Fang-Fang Jian

**Affiliations:** a Microscale Science Institute, Biology Department, Weifang University, Weifang 261061, People’s Republic of China; b Microscale Science Institute, Weifang University, Weifang 261061, People’s Republic of China

## Abstract

The title compound, C_8_H_10_N_2_O_2_S, was prepared by the reaction of 2-cyano-3,3-bis­(methyl­sulfan­yl)acrylate and 2-amino­ethanethiol at 350 K. The mol­ecular structure and packing are stabilized by N—H⋯O hydrogen-bond inter­actions. All the non-H atoms are nearly in the same plane with the maximum deviation being 0.08 Å.

## Related literature

For biological properties of compounds containing thia­zolidine groups, see: Huang & Shi (1990 ▶); Iwata *et al.* (1988 ▶). For related compounds, see: Schroth *et al.* (1997 ▶).
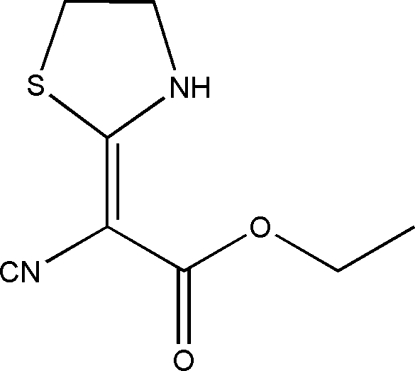


## Experimental

### 

#### Crystal data

C_8_H_10_N_2_O_2_S
*M* *_r_* = 198.24
Monoclinic, 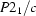

*a* = 4.0676 (8) Å
*b* = 15.460 (3) Å
*c* = 14.581 (3) Å
β = 90.03 (3)°
*V* = 916.9 (3) Å^3^
*Z* = 4
Mo *K*α radiation
μ = 0.32 mm^−1^
*T* = 293 (2) K
0.20 × 0.12 × 0.09 mm

#### Data collection

Bruker SMART CCD area-detector diffractometer
Absorption correction: none
6717 measured reflections
1577 independent reflections
1484 reflections with *I* > 2σ(*I*)
*R*_int_ = 0.025

#### Refinement

*R*[*F* ^2^ > 2σ(*F* ^2^)] = 0.051
*wR*(*F* ^2^) = 0.112
*S* = 1.24
1577 reflections
119 parameters
H-atom parameters constrained
Δρ_max_ = 0.26 e Å^−3^
Δρ_min_ = −0.33 e Å^−3^

### 

Data collection: *SMART* (Bruker, 1997 ▶); cell refinement: *SAINT* (Bruker, 1997 ▶); data reduction: *SAINT*; program(s) used to solve structure: *SHELXS97* (Sheldrick, 2008 ▶); program(s) used to refine structure: *SHELXL97* (Sheldrick, 2008 ▶); molecular graphics: *SHELXTL* (Sheldrick, 2008 ▶); software used to prepare material for publication: *SHELXTL*.

## Supplementary Material

Crystal structure: contains datablocks global, I. DOI: 10.1107/S1600536808032984/at2647sup1.cif

Structure factors: contains datablocks I. DOI: 10.1107/S1600536808032984/at2647Isup2.hkl

Additional supplementary materials:  crystallographic information; 3D view; checkCIF report

## Figures and Tables

**Table 1:** Hydrogen-bond geometry (Å, °)

*D*—H⋯*A*	*D*—H	H⋯*A*	*D*⋯*A*	*D*—H⋯*A*
N1—H1*A*⋯O1^i^	0.86	2.33	2.955 (3)	129
N1—H1*A*⋯O1	0.86	2.12	2.723 (3)	127

Symmetry code: (i) 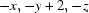.

**Fig. 1.**
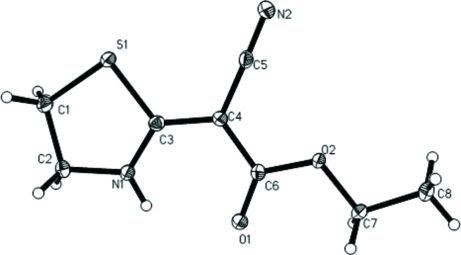
The molecular structure of the title compound with the atom-labeling scheme. Displacement ellipsoids are drawn at the 30% probability level.

## supplementary crystallographic information

### Comment

Thiazolidine is an important kind of group in organic chemistry. Many compounds containing thiazolidine groups possess a broad spectrum of biological activities (Iwata *et al.*, 1988; Huang & Shi, 1990). Here, we report the crystal structure of the title compound (I).

In the crystal structure of (I) (Fig. 1), the torsion angle formed by the N1, C3, S1 and C1 is 4.5 (3)°. All the non-H atoms are nearly the same plane with the maximum deviation of atoms being 0.08 Å. The C—S bond lengths of 1.745 (3) and 1.806 (3) Å are in agreement with those observed before (Schroth *et al.*, 1997). In the crystal structure, there are N—H···O hydrogen-bond interactions to stabilize the crystal structure (Table 12).

### Experimental

A mixture of ethyl 2-cyano-3,3-bis(methylthio)acrylate 4 mmol (0.87 g) and 2-amino-ethanethiol (0.32 g, 4.1 mmol) is refluxed in absolute EtOH (25 ml) for 4 h. On cooling, the product crystallized and is filtered, and recrystallized from absolute EtOH [yield 0.67 g (85%)]. Single crystals suitable for X-ray measurements were obtained by recrystallization from ethanol at room temperature.

### Refinement

H atoms were positioned geometrically and allowed to ride on their parent atoms, with N—H and C—H distances of 0.86 and 0.93–0.96 Å, respectively, and with *U*_iso_(H) = 1.2 or 1.5*U*_eq_ of the parent atoms.

### Crystal data

**Table:**

C_8_H_10_N_2_O_2_S	*F*(000) = 416
*M**_r_* = 198.24	*D*_x_ = 1.436 Mg m^−^^3^
Monoclinic, *P*2_1_/*c*	Mo *K*α radiation, λ = 0.71073 Å
Hall symbol: -P 2ybc	Cell parameters from 2422 reflections
*a* = 4.0676 (8) Å	θ = 2.3–25.1°
*b* = 15.460 (3) Å	µ = 0.32 mm^−^^1^
*c* = 14.581 (3) Å	*T* = 293 K
β = 90.03 (3)°	Needle, colourless
*V* = 916.9 (3) Å^3^	0.20 × 0.12 × 0.09 mm
*Z* = 4	

### Data collection

**Table:**

Bruker SMART CCD area-detector diffractometer	1484 reflections with *I* > 2σ(*I*)
Radiation source: fine-focus sealed tube	*R*_int_ = 0.025
graphite	θ_max_ = 25.0°, θ_min_ = 3.1°
φ and ω scans	*h* = −4→4
6717 measured reflections	*k* = −18→18
1577 independent reflections	*l* = −17→17

### Refinement

**Table:**

Refinement on *F*^2^	Secondary atom site location: difference Fourier map
Least-squares matrix: full	Hydrogen site location: inferred from neighbouring sites
*R*[*F*^2^ > 2σ(*F*^2^)] = 0.051	H-atom parameters constrained
*wR*(*F*^2^) = 0.112	*w* = 1/[σ^2^(*F*_o_^2^) + 2.059*P*] where *P* = (*F*_o_^2^ + 2*F*_c_^2^)/3
*S* = 1.24	(Δ/σ)_max_ < 0.001
1577 reflections	Δρ_max_ = 0.26 e Å^−^^3^
119 parameters	Δρ_min_ = −0.33 e Å^−^^3^
0 restraints	Extinction correction: SHELXL97 (Sheldrick, 2008), Fc^*^=kFc[1+0.001xFc^2^λ^3^/sin(2θ)]^-1/4^
Primary atom site location: structure-invariant direct methods	Extinction coefficient: 0.041 (3)

### Special details

**Table:**

Geometry. All e.s.d.'s (except the e.s.d. in the dihedral angle between two l.s. planes) are estimated using the full covariance matrix. The cell e.s.d.'s are taken into account individually in the estimation of e.s.d.'s in distances, angles and torsion angles; correlations between e.s.d.'s in cell parameters are only used when they are defined by crystal symmetry. An approximate (isotropic) treatment of cell e.s.d.'s is used for estimating e.s.d.'s involving l.s. planes.
Refinement. Refinement of *F*^2^ against ALL reflections. The weighted *R*-factor *wR* and goodness of fit *S* are based on *F*^2^, conventional *R*-factors *R* are based on *F*, with *F* set to zero for negative *F*^2^. The threshold expression of *F*^2^ > σ(*F*^2^) is used only for calculating *R*-factors(gt) *etc*. and is not relevant to the choice of reflections for refinement. *R*-factors based on *F*^2^ are statistically about twice as large as those based on *F*, and *R*- factors based on ALL data will be even larger.

### Fractional atomic coordinates and isotropic or equivalent isotropic displacement parameters (Å^2^)

**Table:**

	*x*	*y*	*z*	*U*_iso_*/*U*_eq_	
S1	0.2265 (2)	0.70109 (5)	0.01523 (5)	0.0285 (3)	
O2	−0.2856 (6)	0.91026 (13)	0.24156 (14)	0.0250 (5)	
O1	−0.1022 (6)	0.97114 (14)	0.11080 (15)	0.0325 (6)	
N2	−0.1952 (7)	0.68892 (17)	0.23236 (18)	0.0304 (7)	
N1	0.1928 (7)	0.86360 (17)	−0.01208 (17)	0.0279 (7)	
H1A	0.1440	0.9171	−0.0037	0.034*	
C3	0.1091 (7)	0.80489 (19)	0.0489 (2)	0.0209 (6)	
C6	−0.1433 (8)	0.9071 (2)	0.1582 (2)	0.0226 (7)	
C7	−0.3933 (9)	0.9959 (2)	0.2710 (2)	0.0270 (7)	
H7A	−0.5582	1.0182	0.2291	0.032*	
H7B	−0.2088	1.0356	0.2717	0.032*	
C1	0.3729 (10)	0.7390 (2)	−0.0946 (2)	0.0337 (8)	
H1B	0.5946	0.7182	−0.1053	0.040*	
H1C	0.2324	0.7174	−0.1433	0.040*	
C4	−0.0547 (8)	0.82003 (19)	0.13177 (19)	0.0213 (7)	
C5	−0.1330 (7)	0.7482 (2)	0.18828 (19)	0.0220 (7)	
C2	0.3686 (8)	0.8366 (2)	−0.0937 (2)	0.0263 (7)	
H2A	0.5914	0.8590	−0.0926	0.032*	
H2B	0.2593	0.8584	−0.1481	0.032*	
C8	−0.5358 (9)	0.9868 (2)	0.3661 (2)	0.0330 (8)	
H8A	−0.6097	1.0423	0.3873	0.049*	
H8B	−0.3703	0.9650	0.4069	0.049*	
H8C	−0.7180	0.9474	0.3645	0.049*	

### Atomic displacement parameters (Å^2^)

**Table:**

	*U*^11^	*U*^22^	*U*^33^	*U*^12^	*U*^13^	*U*^23^
S1	0.0436 (5)	0.0177 (4)	0.0241 (4)	0.0040 (4)	0.0121 (3)	0.0002 (3)
O2	0.0389 (13)	0.0167 (10)	0.0192 (10)	0.0020 (9)	0.0100 (9)	−0.0013 (8)
O1	0.0527 (15)	0.0197 (11)	0.0251 (12)	0.0021 (11)	0.0141 (11)	0.0033 (9)
N2	0.0444 (17)	0.0255 (14)	0.0215 (14)	0.0008 (13)	0.0075 (12)	0.0017 (12)
N1	0.0421 (17)	0.0182 (13)	0.0234 (14)	0.0058 (12)	0.0128 (12)	0.0028 (10)
C3	0.0237 (15)	0.0194 (14)	0.0197 (15)	0.0003 (13)	−0.0012 (12)	0.0001 (12)
C6	0.0272 (17)	0.0223 (16)	0.0183 (14)	−0.0010 (13)	0.0039 (12)	−0.0021 (12)
C7	0.0385 (19)	0.0182 (15)	0.0244 (16)	0.0012 (14)	0.0057 (14)	−0.0035 (12)
C1	0.051 (2)	0.0258 (17)	0.0245 (17)	0.0088 (16)	0.0135 (15)	0.0034 (13)
C4	0.0274 (16)	0.0180 (15)	0.0183 (14)	−0.0005 (13)	0.0030 (12)	0.0010 (11)
C5	0.0254 (16)	0.0240 (16)	0.0166 (14)	0.0029 (13)	0.0033 (12)	−0.0030 (12)
C2	0.0312 (17)	0.0266 (17)	0.0212 (15)	0.0000 (14)	0.0076 (13)	0.0001 (13)
C8	0.045 (2)	0.0264 (17)	0.0275 (17)	0.0059 (16)	0.0109 (15)	−0.0042 (13)

### Geometric parameters (Å, °)

**Table:**

S1—C3	1.745 (3)	C7—C8	1.510 (4)
S1—C1	1.806 (3)	C7—H7A	0.9700
O2—C6	1.348 (3)	C7—H7B	0.9700
O2—C7	1.459 (4)	C1—C2	1.510 (4)
O1—C6	1.218 (4)	C1—H1B	0.9700
N2—C5	1.147 (4)	C1—H1C	0.9700
N1—C3	1.315 (4)	C4—C5	1.420 (4)
N1—C2	1.450 (4)	C2—H2A	0.9700
N1—H1A	0.8600	C2—H2B	0.9700
C3—C4	1.400 (4)	C8—H8A	0.9600
C6—O1	1.218 (4)	C8—H8B	0.9600
C6—C4	1.446 (4)	C8—H8C	0.9600
			
C3—S1—C1	92.39 (14)	S1—C1—H1B	110.1
C6—O2—C7	115.3 (2)	C2—C1—H1C	110.1
C3—N1—C2	119.0 (3)	S1—C1—H1C	110.1
C3—N1—H1A	120.5	H1B—C1—H1C	108.4
C2—N1—H1A	120.5	C3—C4—C5	118.5 (3)
N1—C3—C4	126.3 (3)	C3—C4—C6	120.3 (3)
N1—C3—S1	111.9 (2)	C5—C4—C6	121.2 (3)
C4—C3—S1	121.8 (2)	N2—C5—C4	178.5 (3)
O1—C6—O2	122.8 (3)	N1—C2—C1	107.5 (2)
O1—C6—O2	122.8 (3)	N1—C2—H2A	110.2
O1—C6—C4	124.8 (3)	C1—C2—H2A	110.2
O1—C6—C4	124.8 (3)	N1—C2—H2B	110.2
O2—C6—C4	112.4 (3)	C1—C2—H2B	110.2
O2—C7—C8	107.5 (2)	H2A—C2—H2B	108.5
O2—C7—H7A	110.2	C7—C8—H8A	109.5
C8—C7—H7A	110.2	C7—C8—H8B	109.5
O2—C7—H7B	110.2	H8A—C8—H8B	109.5
C8—C7—H7B	110.2	C7—C8—H8C	109.5
H7A—C7—H7B	108.5	H8A—C8—H8C	109.5
C2—C1—S1	108.2 (2)	H8B—C8—H8C	109.5
C2—C1—H1B	110.1		
			
C2—N1—C3—C4	178.7 (3)	S1—C3—C4—C5	−1.6 (4)
C2—N1—C3—S1	−1.4 (4)	N1—C3—C4—C6	−1.1 (5)
C1—S1—C3—N1	−4.5 (3)	S1—C3—C4—C6	178.9 (2)
C1—S1—C3—C4	175.4 (3)	O1—C6—C4—C3	4.2 (5)
O1—O1—C6—O2	0.0 (3)	O1—C6—C4—C3	4.2 (5)
O1—O1—C6—C4	0.0 (3)	O2—C6—C4—C3	−177.2 (3)
C7—O2—C6—O1	1.0 (4)	O1—C6—C4—C5	−175.3 (3)
C7—O2—C6—O1	1.0 (4)	O1—C6—C4—C5	−175.3 (3)
C7—O2—C6—C4	−177.6 (3)	O2—C6—C4—C5	3.3 (4)
C6—O2—C7—C8	−178.4 (3)	C3—N1—C2—C1	8.0 (4)
C3—S1—C1—C2	8.7 (3)	S1—C1—C2—N1	−10.3 (4)
N1—C3—C4—C5	178.4 (3)		

### Hydrogen-bond geometry (Å, °)

**Table:**

*D*—H···*A*	*D*—H	H···*A*	*D*···*A*	*D*—H···*A*
N1—H1A···O1^i^	0.86	2.33	2.955 (3)	129
N1—H1A···O1	0.86	2.12	2.723 (3)	127

Symmetry codes: (i) −*x*, −*y*+2, −*z*.
